# Genetic Profiling and Performance Optimization in Elite Combat Sport Athletes: A Cross-Sectional Study Based on Total Genetic Score Analysis

**DOI:** 10.3390/genes16040461

**Published:** 2025-04-17

**Authors:** Andrea Pagliaro, Anna Alioto, Alessia Boatta, Giuseppe Messina, Patrik Drid, Paolo Milazzo, Cristina Cortis, Andrea Fusco, Sonya Vasto, Patrizia Proia, Sara Baldassano

**Affiliations:** 1 Department of Psychology, Educational Science and Human Movement, Sport and Exercise Sciences Research Unit, University of Palermo, 90128 Palermo, Italy; andrea.pagliaro@unipa.it (A.P.); anna.alioto@unipa.it (A.A.); alessia.boatta@uniroma5.it (A.B.); paolo.milazzo86@gmail.com (P.M.); 2 Department of Human Sciences and Promotion of the Quality of Life, San Raffaele University, 00166 Rome, Italy; giuseppe.messina@uniroma5.it; 3 Institute for Biomedical Research and Innovation, National Research Council (CNR), 90146 Palermo, Italy; 4 Faculty of Sport and Physical Education, University of Novi Sad, 21000 Novi Sad, Serbia; patrikdrid@uns.ac.rs; 5 Department of Human Sciences, Society and Health, University of Cassino and Lazio Meridionale, 03043 Cassino, Italy; c.cortis@unicas.it; 6 Department of Medicine and Aging Sciences, University “G. d’Annunzio” of Chieti-Pescara, 66100 Chieti, Italy; andrea.fusco@unich.it; 7 Department of Biological Chemical and Pharmaceutical Sciences and Technologies (STEBICEF), University of Palermo, 90128 Palermo, Italy; sonya.vasto@unipa.it (S.V.); sara.baldassano@unipa.it (S.B.); 8 Euro-Mediterranean Institute of Science and Technology (IEMEST), University of Palermo, 90139 Palermo, Italy

**Keywords:** point-fighting, performance-enhancing polymorphism, total genetic score, personalized training

## Abstract

Background/Objectives: The interplay between genetics and athletic performance has garnered significant attention, particularly regarding performance-enhancing polymorphisms (PEPs) and their role in determining key traits that are critical for athletic success. Therefore, this study investigates the genetic predispositions related to peroxisome proliferator-activated receptor alpha (PPARα), angiotensin converting enzyme (ACE), and creatine kinase muscle-type (CKM) gene variants and their potential influence on elite point-fighting (PF) athletes. Methods: A total of 24 elite PF athletes (12 women and 12 men; age = 22.1 ± 5.8 years; body mass = 66.1 ± 15.4 kg; and height = 173.0 ± 9.5 cm, BMI = 21.8 ± 3.2 kg·m^−2^) participated in the study. Saliva samples were collected for DNA extraction and genotyping, analyzing the prevalence of key genetic markers, including the D allele and ID genotype for the ACE variant, the G allele and GG genotype for PPARα, and the A allele and AA genotype for CKM. Results: Genotyping revealed a high prevalence of key genetic markers among participants, with the D allele (58.33%) and ID genotype (66.67%) for the ACE variant, the G allele (77.08%) and GG genotype (54.17%) for PPARα, and the A allele (77.08%) with an AA genotype (62.50%) for CKM. The Total Genetic Score (TGS) analysis indicated a mixed-oriented genetic predisposition across the sample. Conclusions: Although PF athletes showed mixed aerobic/anaerobic genetic profiles, their training routines were primarily strength-oriented, suggesting a possible misalignment between genetic predispositions and their current training approach. These findings offer preliminary insights into the genetic characteristics of elite PF athletes and may inform future investigations into the potential role of genetic information in guiding training strategies.

## 1. Introduction

Physical activity’s positive effects on healthy and unhealthy individuals are well-documented, demonstrating the adaptation of biological systems to stress, such as the circulatory, respiratory, and muscular systems [1]. These adaptations directly influence athletic performance and the ability to excel in specific physical skills [2]. Training induces modifications at the tissue and cellular levels, which are influenced by variations in local gene expression [3]. Specific genetic variants play a pivotal role in determining excellence in traits such as speed, muscle strength, injury predisposition, and emotional control. Recently, the interest in the role of genetics in sports performance has grown, with studies exploring how genetic variations influence physical abilities, endurance, strength, and psychological traits [4].

Performance-enhancing polymorphisms (PEPs) are genetic variations that influence athletic traits, such as endurance, muscle strength, power, flexibility, and other critical components of performance [5]. Understanding these polymorphisms provides valuable insights for tailoring training programs to optimize individual genetic potential, including training adaptations and dietary strategies aligned with genetic predispositions [6]. To identify these polymorphisms, Total Genetic Score (TGS) has emerged as an effective tool for evaluating genetic predisposition while minimizing the need for an extensive number of polymorphisms [7]. By quantifying the combined effect of multiple PEPs, TGS streamlines the assessment of genetic profiles, enabling efficient comparisons and personalized recommendations. Athletes with training regimens aligned with their genetic predispositions achieve superior results compared to those whose training is mismatched with their genetic profiles [8].

Specific genes such as the Angiotensin I Converting Enzyme (ACE), Peroxisome Proliferator-Activated Receptors (PPARα), and Creatine Kinase Muscle-Type (CKM), have been identified as critical contributors to sports performance. The ACE gene, through its I/D polymorphism, influences endurance and strength: the “I” allele is linked to endurance activities like long-distance running, while the D allele correlates with strength-focused disciplines such as weightlifting. Similarly, PPARα, through the C/G polymorphism, affects lipid metabolism, inflammation, and tissue repair, with the G allele favoring endurance due to its association with slow-twitch muscle fibers and aerobic capacity, and the C allele favoring power-based sports [9,10,11]. The CKM gene, involved in muscle energy metabolism, demonstrates a similar dual influence, with the A allele associated with higher aerobic capacity and the G allele linked to greater muscle strength and power [12,13].

This study focuses on the importance of the genetic background in elite athletes, emphasizing the interaction between genetic and environmental factors in determining athletic success. By focusing on Point Fighting (PF) athletes, this research aims to fill a gap in the literature, exploring their genetic predispositions to support the development of tailored training strategies [14]. These findings are expected to advance scientific knowledge and provide practical applications for optimizing individual performance in this discipline.

## 2. Materials and Methods

### 2.1. Study Design

We aimed to focus on the ACE, PPARα, and CKM due to their well-established roles in key physical attributes relevant to PF, since these genes are directly involved in energy metabolism, muscular power, and endurance-crucial elements in this sport. Specifically, PPARα influences lipid and glucose metabolism and is expressed in metabolically active tissues; the C allele has been associated with power-oriented phenotypes, while the G allele favors endurance [12,15,16]. The ACE I/D polymorphism modulates enzyme expression impacting vasodilation and oxygen delivery, with the I allele linked to endurance and the D allele to power [17,18,19]. Lastly, the CKM gene encodes muscle creatine kinase, which is vital for muscle energy homeostasis [20]. This gene selection supports a targeted understanding of the physiological mechanisms relevant to PF and the development of discipline-specific training personalization.

### 2.2. Participants

A total of 24 PF elite athletes (12 women and 12 men) were enrolled in this cross-sectional study following the STROBE checklist recommendations (Supplementary Material). The anthropometric characteristics of the participants are shown in Table 1.

This study included elite athletes selected based on their performance in top-level international competitions organized by the WAKO federation (https://www.wako.sport/, accessed on 3 April 2025). Specifically, during 2018–2019, all participants had achieved a podium at European and World Championships in their respective weight categories. This selection resulted in a homogeneous cohort composed of predominantly European athletes, significantly limiting ancestral variability within the sample. Several studies indicate that within cohorts of European athletes, the distribution of ACTN3 R577X and ACE I/D polymorphisms tends to be stable and comparable, with limited influence of genetic stratification [21,22,23]. Furthermore, the performance-oriented polygenic profile (ACE, ACTN3, PPARA, and CKM) appears to be consistent across European athlete groups, as shown by Ruiz et al. [24].

The inclusion criteria specified that participants had to be high-level athletes with international experience, have passed a selection process based on their competitive results, and be free from musculoskeletal injuries that could impair their performance during the study. A medical doctor checked these conditions before the official call-up was accepted. In combat sports, disabling injuries—such as fractures or severe sprains of the upper or lower limbs—can significantly compromise athletic performance and safety during combat. For this reason, the selected athletes underwent a medical examination and declared that they were in good physical condition, a requirement for participation in the fights scheduled during the camp. Moreover, the practical and competitive nature of the training camp, which included actual sparring sessions, made any physical limitations immediately apparent, effectively ruling out any false declarations or attempts to hide injuries. Selection was carried out using an elite index, specially developed to identify high-level athletes. This index was calculated based on the WAKO Current Ranking, an official sports identity card provided by the International WAKO federation, which can be consulted online and collects the results obtained by each athlete in major international competitions. Only the athletes who, in major competitions, had reached the podium and finished consistently in the top three were considered. Then, based on these results, the selected athletes were invited to an official training camp with the Italian National Kickboxing Team, aiming to determine the shortlist that would represent Italy at the European Championships. Only those athletes invited to the camp and thus officially recognized as part of the national elite were included in the study. Those who did not meet these criteria were excluded at a preliminary stage. Consequently, all recruited athletes fully met the inclusion criteria and no further exclusion criteria were necessary after their recruitment.

After providing informed consent, all athletes completed a questionnaire so that we could collect anthropometric data and information about their weekly training habits. In this questionnaire (provided in the Supplementary Materials section), they were asked about the duration and frequency of their daily technical, aerobic, and resistance workouts. The questionnaire was administered via an interview method where we explained to the athletes what was meant by aerobic (running, swimming, and cycling) and resistance workouts (free weight, body weight, and machines) following the American College of Sports Medicine guidelines [25]. A saliva sample was then collected from each participant for genetic analysis to determine the allelic distribution of the analyzed genes. Finally, a standardized TGS was applied to the identified genotype to assess whether the athletes’ genetic profiles were more oriented toward power or aerobic endurance. The study was conducted according to the Declaration of Helsinki and was approved by the ethics committee of the University of Novi Sad, Serbia (ref. no. 46-06-02/2020-2).

### 2.3. Total Genetic Score

The Total Genetic Score (TGS) we have chosen to apply for data analysis was power-oriented. Therefore, a score of “2” was attributed when the polymorphism was homozygous for the allele predisposing to power activities, “1” was attributed if it was heterozygous, and “0” when the polymorphism was homozygous for the allele predisposing to aerobic activities. The sum of the scores given to the polymorphism of each gene gives us the genotype score (GS) (maximum score = 6) [26].

Table 2 shows the association between the GS of each polymorphism and the metabolic impact.

After identifying the GS for each gene polymorphism, the score was reformulated on a centesimal basis, obtaining the TGS. The formula used was TGS = GS * 100/6 according to the formula which was validated by Hughes and colleagues [27]. For example, subjects having the following genetic profile: ACE DD (2), PPARA CG (1) CKM AA (0) have a GS = 3 and therefore a TGS = 50% power-oriented.

### 2.4. Statistical Analysis

Descriptive statistical analysis was performed with SPSS Statistics 29 software and “Jamovi” software version “2.3.21.0”. Percentages were used to analyze the participant’s allelic distribution in the three genes. Data were expressed as percentages, means, and standard deviations. The normality of continuous variables (e.g., age, anthropometric data, and training duration) was assessed using the Shapiro–Wilk test. The post hoc power analysis was conducted using G*Power software version ‘’3.1.9.6’’. A *t*-test analysis was performed with an effect size (d) of 0.73, an α error probability of 0.05, and a total sample size of 24, resulting in a statistical power (1 − β) of 0.72. This indicates a 72% probability of detecting a true effect, which is slightly below the recommended threshold of 80% or higher. A *p*-value < 0.05 was considered statistically significant.

### 2.5. DNA Extraction and Genotyping

From the saliva samples collected and stored at −20°, genomic DNA extraction was performed using a “Saliva DNA ISO isolation kit” from Norgen Biotek (Thorold, ON, Canada) according to the procedure already described in our previous study [28]. Details regarding the protocol used for genotyping by polymerase chain reaction (PCR) are provided in Table 3.

As described in Table 4, two of the polymorphisms analyzed required enzymatic digestion, and the fragments obtained were highlighted by the gel electrophoresis method [29].

## 3. Results

The genotyping results showed for the ACE gene a predominance of the ID genotype (66.67%), followed by the DD genotype (25%) and the II genotype (8.33%), with a higher expression frequency of the D allele versus the I allele (58.33% vs. 41.67%).

Regarding PPARα polymorphisms, we highlighted a prevalence of the GG genotype (54.17%), followed by the CG genotype (45.83%); no CC genotype was found. The G allele was much more common (77.08%) compared with 22.92% for the C allele.

As a concern, the CKM gene variant, the AA genotype, was found with a 62.50% frequency compared to the AG genotype (29.17%) and the GG genotype (8.33%). Analyzing the frequency of expression of the alleles, the A allele was the most common (77.08%) compared with the G allele (22.92%).

**Figure 1:**
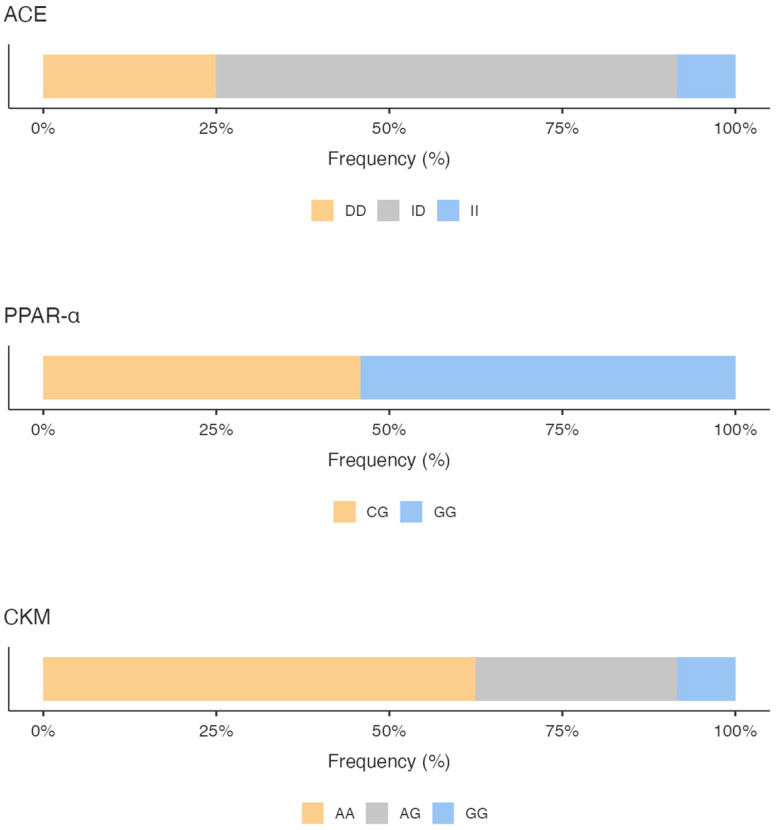
Percentage of the genotype distribution for the three gene variants. ACE: Angiotensin I Converting Enzyme; PPARα: Peroxisome Proliferator-Activated Receptors; CKM: Creatine Kinase Muscle-Type.

**Figure 2:**
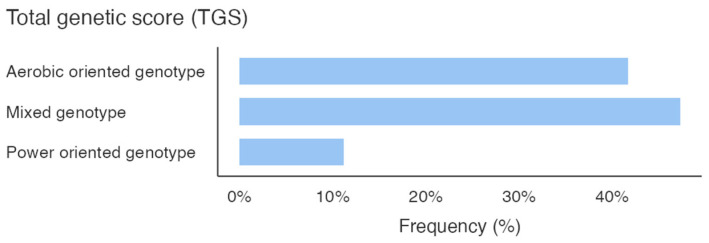
Total genetic score (TGS).

Figure 1 shows the percentage of the genotype distribution for the three gene variants in the whole sample; different colors emphasize the genotype that predisposes the athletes to the specific activity. Additionally, considering the total GS calculated by summing the GS of the allelic distribution for all three genes, it is pointed out that the sample scored a higher mixed-oriented genotype of 47% (Figure 2). The athlete’s survey results showed that they spent an average of 367.3 ± 153.1 min weekly in technique training, 188.5 ± 122.3 min in strength training, and 134.8 ± 95.5 min in aerobic training (Figure 2).

## 4. Discussion

This study identified a high prevalence of mixed aerobic/anaerobic genetic profiles in elite PF athletes. Genotyping revealed dominant frequencies of the D allele (58.33%) and ID genotype (66.67%) for the ACE gene, the G allele (77.08%) and GG genotype (54.17%) for the PPARα gene, and the A allele (77.08%) and AA genotype (62.50%) for the CKM gene. The TGS indicated that 47% of the sample showed a mixed-oriented genetic predisposition. Conversely, athletes’ training routines showed a greater emphasis on strength (188.5 ± 122.3 min/week) over aerobic (134.8 ± 95.5 min/week) training, suggesting a possible misalignment between genetic traits and current training practices. The novelty of our study lies in its preliminary characterization of the genetic background of elite PF athletes by using the TGS. These findings may contribute to future efforts aimed at exploring how genetic predispositions could inform more individualized and scientifically grounded training approaches.

Previous research has established that specific genetic variants significantly contribute to variations in power, endurance, and mixed activities. In this regard, the study by John and colleagues [19] demonstrated the critical role of the ACE I/D polymorphism in physical performance [30]. The ACE I allele, associated with lower ACE levels, facilitates vasodilation and increased oxygenated blood flow to active muscles [20], conferring an advantage in endurance activities [31]. Conversely, the ACE D allele correlates with greater strength, basal muscle volume, and a higher percentage of fast-twitch muscle fibers, favoring power sports [9].

Our findings revealed a greater prevalence of the D allele (58.33%) among elite PF athletes, which has been previously associated with anaerobic-oriented performance traits. This suggests a genetic predisposition favoring power and strength capabilities, which are crucial to the high-intensity, intermittent demands of PF. The ID genotype (66.67%) was predominant, suggesting a mixed capacity for both aerobic and anaerobic performance [32]. These results represent a preliminary insight into the potential genetic characteristics of elite PF athletes, also suggesting that further studies could explore how genetic predispositions relate to current training practices.

Regarding the PPARα polymorphism, the G allele—associated with endurance—was the most frequent in our cohort (77.08%), aligning with findings from Kurtulus and colleagues [12]. This observation highlights the potential relevance of aerobic capacity in a sport traditionally considered anaerobic-dominant. Similarly, for the CKM variant, while a previous study [22] has linked the G allele to power and strength qualities, our study found a higher prevalence of the A allele (62.50%), indicative of a genetic orientation toward aerobic performance [33]. The relatively low presence of power-oriented alleles (PPARα C and CKM G) at approximately 22.92% may reflect a broader genetic predisposition toward mixed or aerobic profiles among these athletes. While these genetic patterns are not conclusive, they suggest that aerobic contributions in PF may be more relevant than usually assumed. Further studies are needed to investigate whether current training regimens adequately address this component.

The athlete surveys data further underscored a preference for strength (188.5 ± 122.3 min per week) over aerobic (134.8 ± 95.5 min per week) training. This training pattern appears only partially aligned with the mixed-oriented genetic profiles observed in our athletes. This imbalance might suggest an underutilization of aerobic capacity, potentially limiting broader performance development. Our sample’s higher frequency of mixed-oriented TGS scores (Figure 2) support the relevance of both aerobic and anaerobic demands in PF athletes. Previous work by Jones and colleagues [8] emphasized the potential benefits of aligning training modalities with genetic predispositions, though such approaches require further experimental validation. In light of our findings, increasing aerobic training components, particularly in athletes with mixed or endurance-oriented genotypes, may represent a valuable approach. These results contribute to the broader understanding of how PF performance could benefit from a more balanced approach (integrating both aerobic and anaerobic elements), and suggest a more versatile physiological profile that may call for greater variability in training focus.

The high frequency of polymorphisms associated with both aerobic and anaerobic performance observed in this cohort of elite athletes challenges the traditional view of PF as a predominantly anaerobic sport. Rather than affirming the sole dominance of anaerobic traits, these findings point to the potential co-relevance of aerobic capacity in this discipline. One possible reason this issue remains debated is the lack of large-scale genetic studies and the heterogeneity of athlete samples considered [30].

Despite these strengths, this study has several limitations. The relatively small sample size (n = 24) may limit statistical power and reduce the generalizability of the findings to the broader population of PF athletes. Moreover, the absence of a comparison group, such as amateur or recreational athletes, makes it difficult to determine whether the observed allele frequencies are unique to elite-level athletes. Although training data were gathered through structured interviews based on standardized definitions from the ACSM guidelines [25], the reliance on self-reported measures introduces the risk of recall bias. Additionally, this study did not include direct performance indicators (e.g., VO_2_max, repeated-sprint ability, or competitive outcomes), which prevents an in-depth examination of how genotypes might relate to actual performances. Furthermore, the genetic analysis was limited to three well-documented performance-related genes (the ACE, PPARα, and CKM), which might not capture the full complexity of the polygenic nature of athletic traits. Variants such as ACTN3, MSTN, or COL5A1, implicated in muscle function, recovery, and injury risk, could provide a more comprehensive picture of the genetic architecture in elite combat sports. Lastly, while a genotype-based training framework is conceptually discussed, no intervention was conducted to test its practical effects. Therefore, the genotype-training alignment remains hypothetical. Future research should aim to include larger cohorts, objective training, performance parameters, and experimental validation to evaluate the actual impact of genotype-informed training strategies.

## 5. Conclusions

This study reveals a high prevalence of mixed aerobic/anaerobic genetic profiles in elite PF athletes, challenging the traditional view of the sport as predominantly anaerobic. In particular, the high proportion of mixed TGS profiles suggests that the physiological demands of PF may be more diverse than previously assumed. These findings contribute to the ongoing discussion about the role of aerobic capacity in combat sports, highlighting the potential mismatch between genetic predispositions and current training emphases.

Although this study did not test any training interventions, the observed genetic patterns point to a promising area for future applied research. The literature [8] has shown that aligning training modalities with genetic profiles can improve physical adaptations. In this context, the TGS may represent a useful tool for better understanding individual predispositions and generating hypotheses about training balance. However, the practical application of TGS-guided strategies remains hypothetical and should be assessed through controlled experimental studies.

## Figures and Tables

**Table 1:** Means and standard deviations (SD) of the participants’ anthropometric characteristics.

Characteristics	Mean ± SD
Age (years)	22.1 ± 5.8
Body mass (kg)	66.1 ± 15.4
Body height (cm)	173.0 ± 9.5
BMI (kg·m^−2^)	21.8 ± 3.2

BMI = body mass index.

**Table 2:** Power-oriented Genotype score (GS) and the association between each polymorphism and sport metabolism requirement.

Gene	Polymorphism	GS Power Oriented	Involved Metabolism
ACE	DD	2	Anaeorobic
	ID	1	Anaerobic/Aerobic
	II	0	Aerobic
PPARα	CC	2	Anaeorobic
	CG	1	Anaerobic/Aerobic
	GG	0	Aerobic
CKM	GG	2	Anaeorobic
	AG	1	Anaerobic/Aerobic
	AA	0	Aerobic

ACE: Angiotensin I Converting Enzyme; PPARα: Peroxisome Proliferator-Activated Receptors; CKM: Creatine Kinase Muscle-Type.

**Table 3:** Protocol details about the gene variant genotyping.

Gene	Primers Sequence	Denaturation	Annealing	Extension	Cycles
ACE	F:5′GCCCTGCAGGTGTCTGCAGCATGT3′	94°30′′	56°45′′	72°1′	35
	R:5′GGATGGCTCTCCCCGCCTTGTCTC3′				
PPARα	F:5′ACAATCACTCCTTAAATATGGTGG3′	94°30′′	59°30′′	72°30′′	35
	R:5′AAGTAGGGACAGACAGGACCAGTA3′				
CKM	F:5′GGGATGCTCAGACTCACAGA3′	94°40′′	53°45′′	72°1′	30
	R:5′AACTTGAATTTAGCCCAACG3′				

ACE: Angiotensin I Converting Enzyme; PPARα: Peroxisome Proliferator-Activated Receptors; CKM: Creatine Kinase Muscle-Type.

**Table 4:** Protocol applied for Enzymatic Digestion of the PCR Products.

Gene	Restriction Enzyme	Digestion	Polymorphism	Fragment Lenght
ACE	Not required	-	DD	319 bp
			II	597 bp
			ID	319 + 597 bp
PPARα	TAQ I	65°	CC	216 + 50 bp
		2:30 h	GG	266 bp
			CG	266 + 216 + 50 bp
CKM	NCO I	37°	GG	359 bp
		2:30 h	AA	206 + 153 bp
			AG	359 + 206 + 153 bp

ACE: Angiotensin I Converting Enzyme; PPARα: Peroxisome Proliferator-Activated Receptors; CKM: Creatine Kinase Muscle-Type.

## Data Availability

The data presented in this study are openly available in https://github.com/ccortis/Dataset/raw/refs/heads/main/Genes_Dataset.xlsx (accessed on 12 March 2025).

## Supplementary Materials

The following supporting information can be downloaded at: https://www.mdpi.com/article/10.3390/genes16040461/s1, Table S1: Questionnaire regarding the duration and frequency of the athletes’ daily technical, aerobic, and resistance workouts. Table S2: STROBE Checklist.
