# Supplementary material for: Genetic Profiling and Performance Optimization in Elite Combat Sport Athletes: A Cross-Sectional Study Based on Total Genetic Score Analysis
Source: Genes (Basel). 2025 Apr 17;16(4):461. doi: 10.3390/genes16040461 (PMC12026721; doi:10.3390/genes16040461)
Supplement: Supplementary file 1 [file genes-16-00461-s001.zip › Table S1_Questionnaire.pdf]

UNIVERSITÀ DEGLI STUDI DI PALERMO

UNIVERSITY OF PALERMO

School of Human Sciences and Cultural Heritage
Degree Course in Sciences and Techniques of Preventive and Adapted Motory Activity and Motory and Sport Activities
Holder of the research Dott.ssa Patrizia Proia-PhD
Professor of Biochemistry
Email: *patrizia.proia@unipa.it*
Tel. 091/23899919 - 3385911303

TITLE OF THE STUDY

“Studio del profilo genetico in atleti Internazionali di Point Fighter”

"The polygenic profile of International Point Fighter Athletes"

SUPPLEMENTARY FORM

SURNAME		*NAME*	
AGE	*GENDER* M / F	*WEIGHT*	*HEIGHT*
COUNTRY	*BELT*	*WEIGHT CATEGORY*	
HOW LONG YOU PRACTICE THIS SPORT?		*BEST RESULT IN CAREER*	

TECHNICAL DAILY WORKOUTS (NUMBER AND DURATION EACH ONE)

MONDAY:	*TUESDAY:*	*WEDNESDAY:*	*THURSDAY:*	*FRIDAY:*	*SATURDAY:*	*SUNDAY:*
1 2	1 2	1 2	1 2	1 2	1 2	1 2
DURATION: 30’ 45’ 60’ 90’ 120’	*DURATION:* 30’ 45’ 60’ 90’ 120’	*DURATION:* 30’ 45’ 60’ 90’ 120’	*DURATION:* 30’ 45’ 60’ 90’ 120’	*DURATION:* 30’ 45’ 60’ 90’ 120’	*DURATION:* 30’ 45’ 60’ 90’ 120’	*DURATION:* 30’ 45’ 60’ 90’ 120’

AEROBIC DAILY WORKOUTS (NUMBER AND DURATION EACH ONE)

MONDAY:	*TUESDAY:*	*WEDNESDAY:*	*THURSDAY:*	*FRIDAY:*	*SATURDAY:*	*SUNDAY:*
1 2	1 2	1 2	1 2	1 2	1 2	1 2
DURATION: 30’ 45’ 60’ 90’ 120’	*DURATION:* 30’ 45’ 60’ 90’ 120’	*DURATION:* 30’ 45’ 60’ 90’ 120’	*DURATION:* 30’ 45’ 60’ 90’ 120’	*DURATION:* 30’ 45’ 60’ 90’ 120’	*DURATION:* 30’ 45’ 60’ 90’ 120’	*DURATION:* 30’ 45’ 60’ 90’ 120’

RESISTANCE DAILY WORKOUTS (NUMBER AND DURATION EACH ONE)

MONDAY:	*TUESDAY:*	*WEDNESDAY:*	*THURSDAY:*	*FRIDAY:*	*SATURDAY:*	*SUNDAY:*
1 2	1 2	1 2	1 2	1 2	1 2	1 2
DURATION: 30’ 45’ 60’ 90’ 120’	*DURATION:* 30’ 45’ 60’ 90’ 120’	*DURATION:* 30’ 45’ 60’ 90’ 120’	*DURATION:* 30’ 45’ 60’ 90’ 120’	*DURATION:* 30’ 45’ 60’ 90’ 120’	*DURATION:* 30’ 45’ 60’ 90’ 120’	*DURATION:* 30’ 45’ 60’ 90’ 120’

Date ____________________ Signature___________________________________

Readable signature of the operator who collected the consent

____
